# Association Between Dietary Fiber Intake and Heart Failure Among Adults: National Health and Nutrition Examination Survey 2009–2018

**DOI:** 10.3389/fcvm.2022.893436

**Published:** 2022-05-17

**Authors:** Hao Zhang, Zhibin Lin, Jun Chen, Daojing Gan, Haobin Zhou, Zhuang Ma, Xianghui Zeng, Yuting Xue, Xiao Wang, Qiong Zhan, Qingchun Zeng, Dingli Xu

**Affiliations:** State Key Laboratory of Organ Failure Research, Department of Cardiology, Nanfang Hospital, Southern Medical University, Guangzhou, China

**Keywords:** heart failure (HF), dietary fiber intake, dose-response, National Health and Nutrition Examination Survey (NHANES), nutrition

## Abstract

**Objective:**

To explore the association between dietary fiber and heart failure (HF).

**Methods:**

Data were collected from the 2009–2018 National Health and Nutrition Examination Survey. Dietary fiber intake data were obtained from two 24-h dietary recall interviews. Logistic regression and restricted cubic spline models were used to explore the association of dietary intakes of total, cereal, fruit, and vegetable fiber with HF prevalence.

**Results:**

A total of 21869 adults were included in this study. After adjusting for multiple confounding factors, the odds ratios (OR) and 95% confidence intervals (CI) for HF was 0.49 (0.28 to 0.87, P for trend = 0.016) for the highest tertile versus lowest tertile of total fiber intake. Similar results were observed for cereal but not fruit and vegetable fiber intake. Dose-response analysis indicated that dietary intake of total and cereal fiber were inversely associated with HF in a linear manner.

**Conclusion:**

Intakes of total and cereal fiber were inversely associated with HF in adults.

## Introduction

Heart failure (HF) is a growing global public health burden, with more than 37.7 million individuals estimated to be affected worldwide (1), and the prevalence of HF is increasing as populations age, and poor lifestyle determinants rise (2). Although advances in treatments and devices for HF have substantially improved the survival and quality of life in patients, the mortality rates remain high, with the 10-year survival rate at only 25% (3). Therefore, it is of great importance to identify modifiable factors for the prevention or delay of HF and its complications.

Dietary fiber, which comprises many different, mainly plant-based, that are not completely digested in the human gut, has become increasingly popular in recent years due to its health benefits. Although nutrition guidelines encourage increased consumption of dietary fiber and a consensus statement from the Heart Failure Society of America recommended plant-based diets for patients with HF (4, 5), most individuals in the United States consume less than half of the recommended levels of daily dietary fiber. Notwithstanding the increase in the daily recommended amount to 30 g, just 13% of men and 4% of women adhere to this recommendation (6). The apparent benefits of dietary fiber intake vary substantially from large relative risk reduction to no benefit depending on the outcome or the geographical region (Europe, the United States, Japan, or China). Emerging evidence from randomized trials and observational studies has shown the potential role of dietary fiber intake in reducing coronary heart disease (7, 8), stroke (8), peripheral vascular disease (9), mortality (10), and cancer (11).

However, previous studies were centered just on the association between dietary fiber and many cardiovascular diseases with no scrutiny of its involvement in HF. Several studies have reported that dietary fiber can improve intestinal flora and risk factors for heart failure (e.g., insulin resistance, inflammation, and metabolic disorders) (12–14), suggesting a potential association between dietary fiber and heart failure. We thus examined the relation between HF prevalence and dietary fiber intake.

## Materials and Methods

### Study Population

The National Health and Nutrition Examination Survey (NHANES) is a nationally representative study to assess the health and nutritional status of the non-institutionalized civilian population in the United States. This survey combines several interviews which were initially executed in participants’ homes, and subsequent health examinations were completed in a mobile examination center (MEC). This database is global and public with data released every 2 years and accessed from their website. The sampling method and data collection details have been published elsewhere (15).

**FIGURE 1:**
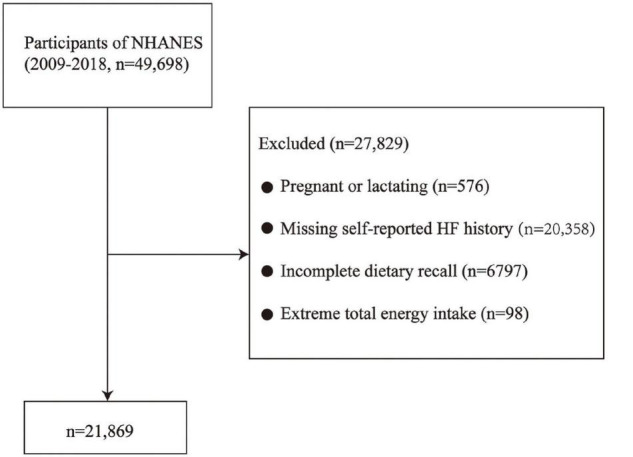
Flow chart of the screening process for the selection of eligible participants. NHANES, National Health and Nutrition Examination Survey.

This study is based on an analysis of data from NHANES cycles between 2009 and 2018. As shown in Figure 1, participants who were pregnant or lactating women (*n* = 576) were excluded. Participants with missing self-reported HF history (*n* = 20,358) or incomplete dietary recall (*n* = 6,797), those documenting extreme total energy intakes, i.e., in women: <500 or >5,000 kcal/day and in men: <500 or >8,000 kcal/day (*n* = 98) were also excluded. Our study included a final total of 21,869 adult participants. As NHANES is a publicly available dataset, this study was exempt from approval by an institutional review board. Informed consent was sourced from the participants prior to the interview and examination steps.

### Dietary Fiber Intake

Dietary fiber intake was assessed by two 24-h dietary recall interviews collected by skilled dietitians. While the MEC was the site of the initial dietary interview, the second interview was collected by telephone after 3 to 10 days. The U.S. Department of Agriculture’s Dietary Research Food and Nutrition Database for Dietary Studies scrutinized the nutrient intakes. Cereal, vegetable, and fruit dietary fiber are calculated by the sum of the corresponding food code in the Individual Foods files. The average 2-day fiber amount was employed to compute the dietary fiber intakes from the two aforementioned interviews and was adjusted to the body weight. In this study, dietary fiber intake was categorized into tertiles.

### Heart Failure

Similar to a prior NHANES study (16), HF was defined as participants who have conducted the audiometric assessment and had a self-reported HF history. The latter entailed an affirmative answer to, “Has a doctor ever diagnosed you with heart failure?”

### Covariates

Data of age, sex, race, education level, family income, and smoking status were collected from household interviews with standardized questionnaires. Bodyweight, height, and blood pressure were obtained when people participated in the physical examinations at a MEC. Plasma glucose and total cholesterol were measured at baseline when the participants provided their blood samples. Body mass index (BMI) was calculated as weight in kilograms (kg) divided by height in meters squared (m^2^). Race classification was Mexican American, other Hispanic, non-Hispanic White, non-Hispanic Black, and other races. Education level was categorized as less than high school, high school or equivalent, or college and above. Family income was classified as <$20,000, $20,000–$55,000, $55,000–$75,000, and ≥$75,000. Smoking status was grouped into “never” (never smoked or less than a hundred cigarettes in life), “current” (more than a hundred cigarettes in life and is also ongoing currently), or “former” (more than a hundred cigarettes in life but currently not smoking). Diabetes was defined as a fasting blood glucose level of ≥7.0 mmol/l, 2-h plasma glucose level of ≥11.1 mmol/l, use of diabetes medications, or self-reported diabetes diagnosis. For hypertension, systolic blood pressure and diastolic blood pressure of ≥130 and ≥80 mmHg, respectively, hypertension medication consumption or self-reported hypertension diagnosis were taken into account. The aggregate daily energy, protein, fat, vitamin A, and vitamin B_6_ from the diet and supplement usage was employed to compute the total energy intake, total protein intake, total fat intake, total vitamin A intake, and total vitamin B_6_ intake, respectively.

### Statistical Analysis

While continuous data were reported as mean and SD, or median with interquartile range, numbers and percentages were employed to express categorical data. Intergroup variation in the former entailed one-way ANOVA and *χ^2^* tests for the latter. Logistic regression models were employed to assess the association between dietary fiber intake and HF prevalence. While Model 1 was unadjusted, model 2 was inclusive of the adjustment for age, sex, and race and model 3 was inclusive of model 2 variables with total energy intake, total protein intake, total fat intake, total vitamin A intake, total vitamin B_6_ intake, BMI, education, annual household income, smoking category, hypertension, diabetes, and total cholesterol (TC) levels. As mentioned above, subsequent to the categorization of the dietary fiber intake as tertiles, the lowest tertile was then employed as the reference group. The odds ratios (ORs) and 95% CIs were calculated. Subgroup analyses ensued for dietary fiber intake and the prevalence of HF by sex, race, hypertension, diabetes, and obesity status. To assess the dose-response association between dietary fiber intake and the prevalence of HF, we fitted a cubic spline regression using the same covariates adjusted in model 3 and located three knots at the 5th, 50th, and 95th. The probabilities here were all two-sided with significance at *P* < 0.05. All analyses were conducted using R version 3.3.3.

## Results

A total of eligible 21,869 participants were included in our study. Mean (SD) age was 50.1 (17.6) years: 10,473 (47.9%) were men, and 9,149 (41.8%) were non-Hispanic White. Population characteristics are presented in Table 1. The HF prevalence is 3.6%. As opposed to non-HF participants, patients with HF were more likely to be older, male, white, highly educated, a current smoker, and were less likely to earn a substantial income and consume high dietary nutrition (such as fiber, protein, fat, vitamin A, and vitamin B_6_) and total energy. They also tended to have lower total cholesterol but higher BMI and higher hypertension and diabetes prevalence.

**TABLE 1:** Characteristics of participants by HF, NHANES 2009–2018.

Characteristics	HF	Non-HF	*P* value
Participants No. (%)	788	21,081	
Male, No. (%)	434 (55.10)	10,039 (47.60)	<0.001
Age, mean (SD),y	67.00 (12.26)	49.42 (17.42)	<0.001
Race, No. (%)			<0.001
Mexican American	68 (8.60)	2,984 (14.20)	
Other Hispanic	70 (8.90)	2,131 (10.10)	
Non-Hispanic White	408 (51.80)	8,741 (41.50)	
Non-Hispanic Black	194 (24.60)	4,517 (21.40)	
Other race	48 (6.10)	2,708 (12.80)	
Income, No. (%)			<0.001
<20,000	76 (45.80)	4,761 (57.90)	
20,000–55,000	46 (27.70)	1,402 (17.10)	
55,000–75,000	25 (15.10)	904 (11.00)	
>75,000	19 (11.40)	1,151 (14.00)	
Education levels, No. (%)			<0.001
Less than high school	256 (32.50)	4,416 (20.90)	
High school or equivalent	210 (26.6)	4,670 (22.20)	
College or above	322 (40.90)	11,995 (56.90)	
Smoking, No. (%)			<0.001
Never	313 (39.70)	12,056 (57.20)	
Former	152 (19.30)	3,974 (18.90)	
Current	323 (41.00)	5,039 (23.90)	
Hypertension No. (%)	715 (90.70)	12,913 (61.30)	<0.001
Diabetes No. (%)	384 (48.70)	3,445 (16.30)	<0.001
TC (SD) (mmol/L)	4.46 (1.12)	4.98 (1.07)	<0.001
Total Fiber intake, mean (SD), (mg/kg/day)	168.42 (101.04)	218.76 (135.36)	<0.001
Cereal fiber intake mean (SD), (mg/kg/day)	91.00 (68.87)	119.17 (88.47)	<0.001
Vegetable fiber intake mean (SD), (mg/kg/day)	43.50 (40.54)	60.62 (64.42)	<0.001
Fruit fiber intake mean (SD), (mg/kg/day)	29.37 (32.91)	39.77 (42.76)	<0.001
BMI, mean (SD) (kg/m2)	32.33 (8.53)	29.37 (7.02)	<0.001
Total energy, mean (SD) (kcal/day)	1783.42 (731.10)	2013.70 (797.37)	<0.001
Total protein, mean (SD) (g/day)	79.75 (34.74)	66.58 (31.06)	<0.001
Total fat, mean (SD) (g/day)	77.45 (37.60)	65.93 (34.94)	<0.001
Total vitamin A, mean (SD) (mg/day)	0.62 (0.53)	0.56 (0.50)	<0.001
Total vitamin B_6_, mean (SD) (mg/day)	2.07 (1.34)	1.69 (1.05)	<0.001

*BMI, body mass index; TC, total cholesterol. Continuous data were expressed as mean and SD. Categorical was expressed as numbers and percentages.*

In univariate logistic regression (Table 2), a consistent association was observed between higher fiber intake (total, cereal, vegetable, and fruit) and lower HF prevalence. These associations remained significant following age, sex, and race adjustments (model 2). After further adjustment for total energy intake, total protein intake, total fat intake, total vitamin A intake, total vitamin B_6_ intake educational level, annual household income, smoking status, hypertension, diabetes, TC, and body mass index (model 3), the ORs of HF were 0.49 (95% CI 0.28–0.87, P_for trend_ = 0.016), 0.59 (95% CI 0.36–0.96, *P*_for trend_ = 0.032), 1.19 (95% CI 0.73–1.92, *P*_for trend_ = 0.439), and 0.56 (95% CI 0.33–0.94, *P*_for trend_ = 0.070) for the highest tertile of the total, cereal, vegetable, and fruit fiber, respectively, as opposed to the lowest tertile. An association remained for the highest tertile of total and cereal fiber with a lowered HF prevalence, while the association between vegetable and fruit fiber with HF was no longer significant.

**TABLE 2:** ORs and 95% CIs for HF according to tertiles of dietary fiber intake, NHANES 2009–2018.

Fiber intake level (g/kg/day)	OR (95%CI)		
	Model 1	Model 2	Model 3
**Total fiber intake**			
T1 (<0.142)	1 (ref)	1 (ref)	1 (ref)
T2 (0.142–0.240)	0.74 (0.63, 0.87)**	0.63 (0.53, 0.75)**	0.58 (0.38, 0.89)*
T3 (>0.240)	0.42 (0.35, 0.51)**	0.39 (0.32, 0.48)**	0.49 (0.28, 0.87)*
P for trend	<0.001	<0.001	0.016
**Cereal fiber intake**			
T1 (<0.069)	1 (ref)	1 (ref)	1 (ref)
T2 (0.069–0.133)	0.77 (0.66, 0.91)**	0.62 (0.53, 0.74)**	0.72 (0.47, 1.11)
T3 (>0.133)	0.44 (0.37, 0.54)**	0.35 (0.29, 0.43)**	0.59 (0.36, 0.96)*
P for trend	<0.001	<0.001	0.032
**Vegetable fiber intake**			
T1 (<0.025)	1 (ref)	1 (ref)	1 (ref)
T2 (0.025–0.064)	0.80 (0.67, 0.96)**	0.77 (0.63, 0.92)**	1.14 (0.74, 1.75)
T3 (>0.064)	0.56 (0.45, 0.68)**	0.57 (0.47, 0.70)**	1.19 (0.73, 1.92)
P for trend	<0.001	<0.001	0.439
**Fruit fiber intake**			
T1 (<0.015)	1 (ref)	1 (ref)	1 (ref)
T2 (0.015–0.043)	0.84 (0.71, 0.99)**	0.65 (0.54, 0.78)**	0.85 (0.55, 1.34)
T3 (>0.043)	0.53 (0.43, 0.65)**	0.36 (0.29, 0.45)**	0.56 (0.33,0.94)*
P for trend	<0.001	<0.001	0.070

OR, odds ratio; CI, confidence interval; T, tertile. Model 1 is unadjusted. Model 2 adjusted for age and sex. Model 3 adjusted for age, sex, race, total energy intake, total protein intake, total fat intake, total vitamin A intake, total vitamin B_6_ intake, BMI, educational level, annual household income, smoking status, hypertension, diabetes and total cholesterol (TC) levels. The lowest tertile of dietary fiber intake was used as the reference group, *P < 0.05; **P < 0.01.

**FIGURE 2:**
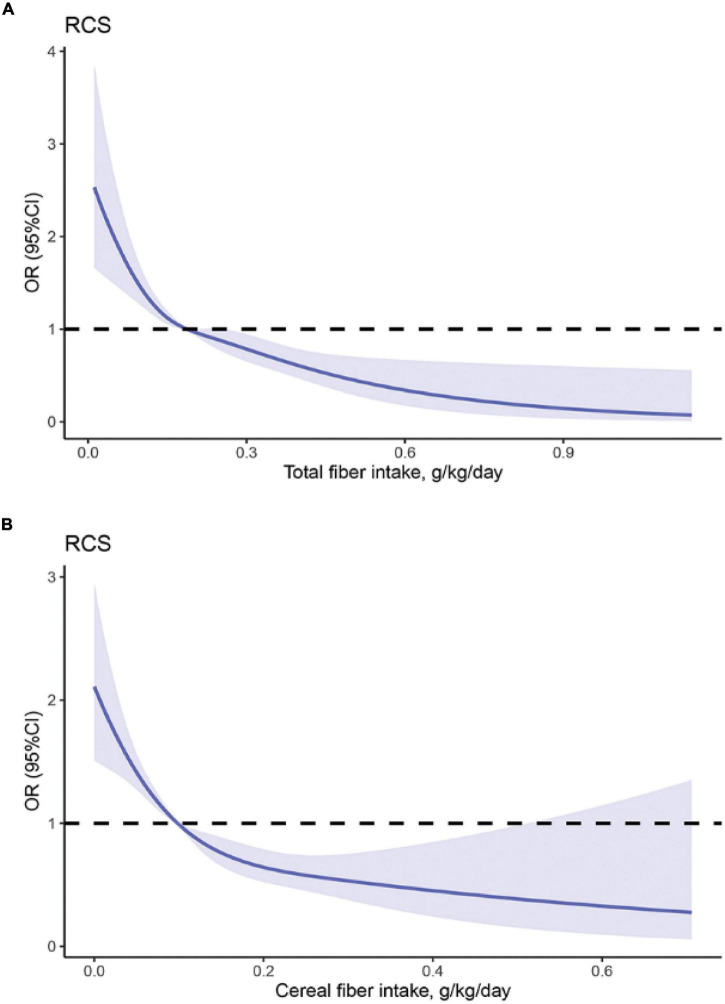
The restricted cubic spline model showed a dose-response relationship between total, cereal fiber intake and HF. **(A)** = Total fiber, P for non-linearity = 0.14; **(B)** Cereal fiber, Pfor non-linearity = 0.27; The restricted cubic splines model adjusted for age, sex, race, total energy intake, total protein intake, total fat intake, total vitamin A intake, total vitamin B_6_ intake, BMI, educational level, annual household income, smoking status, hypertension, diabetes and total cholesterol (TC) levels. The solid line and dashed line represent the estimated ORs and the corresponding 95% confidence intervals, respectively. OR, odds ratio.

The dose-response relationship analysis between total, cereal fiber intake, and HF is shown in Figure 2. A linear relationship was observed between total dietary fiber intake and HF (*P* for non-linearity = 0.14). HF prevalence significantly decreased with the increase in total fiber intake. This linear inverse association was also observed for the cereal fiber intakes and HF prevalence (*P* for non-linearity = 0.27). The dose-response relationship analysis between vegetable and fruit fiber with HF was not performed as no significant association was observed in the multivariable logistic regression analysis (model 3).

The association between dietary fiber intake and HF in subgroup analyses was displayed in Table 3. Subsequent to adjustment for the same covariates in model 3, the subgroup analysis shows that the intake of total and cereal fiber has no significant interaction with sex, race, hypertension, diabetes, and obesity status.

**TABLE 3:** ORs and 95% CIs for HF according to tertiles of total fiber intake, stratified by sex, race, hypertension, diabetes, and obese status, NHANES 2009–2018.

Subgroup	Fiber intake level	Total fiber intake		Cereal fiber intake	
		OR (95%CI)	P for interaction	OR (95%CI)	P for interaction
Sex			0.43		0.35
Male	T1	1		1	
	T2	0.51 (0.28, 0.95)		0.73 (0.40, 1.33)	
	T3	0.58 (0.28, 1.24)		0.59 (0.30, 1.16)	
Female	T1	1		1	
	T2	0.71 (0.38, 1.34)		0.74 (0.40, 1.39)	
	T3	0.44 (0.18, 1.11)		0.66 (0.32, 1.38)	
Race			0.68		0.63
Mexican American	T1	1		1	
	T2	0.91 (0.18, 4.58)		0.17 (0.03, 1.19)	
	T3	0.55 (0.08, 4.02)		0.43 (0.08, 2.39)	
Other Hispanic	T1	1		1	
	T2	1.26 (0.26, 6.07)		0.19 (0.03, 1.36)	
	T3	1.15 (0.16, 8.59)		1.47 (0.24, 9.09)	
Non-Hispanic White	T1	1		1	
	T2	0.31 (0.17, 0.58)		0.69 (0.39, 1.22)	
	T3	0.40 (0.19, 0.86)		0.41 (0.21, 0.80)	
Non-Hispanic Black	T1	1		1	
	T2	1.77 (0.70, 4.43)		1.08 (0.45, 2.59)	
	T3	0.44 (0.08, 2.35)		1.11 (0.37, 3.30)	
Other race	T1	1		1	
	T2	1.51 (0.72, 6.76)		1.18 (0.61, 4.51)	
	T3	0.42 (0.06, 4.08)		0.95 (0.23, 2.74)	
BMI			0.58		0.63
<25	T1	1		1	
	T2	0.50 (0.18, 1.41)		1.03 (0.24, 4.31)	
	T3	0.27 (0.08, 0.92)		0.90 (0.23, 3.62)	
25–30	T1	1		1	
	T2	1.14 (0.46, 2.85)		0.66 (0.28, 1.59)	
	T3	0.68 (0.22, 2.11)		0.33 (0.13, 0.86)	
>30	T1	1		1	
	T2	0.40 (0.22, 0.75)		0.70 (0.40, 1.21)	
	T3	0.75 (0.33, 1.73)		0.76 (0.39, 1.51)	
Hypertension			0.91		0.70
Yes	T1	1		1	
	T2	0.57 (0.36, 0.88)		0.68 (0.44, 1.06)	
	T3	1.29 (0.09, 19.67)		0.60 (0.36, 0.99)	
No	T1	1		1	
	T2	0.57 (0.05, 6.35)		1.64 (0.25, 10.79)	
	T3	0.38 (0.03, 4.91)		0.13 (0.06, 2.36)	
Diabetes			0.14		0.44
Yes	T1	1		1	
	T2	0.57 (0.29, 1.15)		1.07 (0.54, 2.13)	
	T3	0.83 (0.34, 2.03)		1.10 (0.51, 2.37)	
No	T1	1		1	
	T2	0.63 (0.36, 1.09)		0.58 (0.33, 1.01)	
	T3	0.39 (0.19, 0.83)		0.41 (0.21, 0.78)	

*BMI, body mass index; OR, odds ratio; CI, confidence interval; T, tertile. Model is adjusted for age, sex, race, total energy intake, total protein intake, total fat intake, total vitamin A intake, total vitamin B_6_ intake, BMI, educational level, annual household income, smoking status, hypertension, diabetes and total cholesterol (TC) levels. The lowest tertile of dietary fiber intake was used as the reference group.*

## Discussion

This study found an association between higher total and cereal fiber intake and lower HF prevalence in adults. Dose-response analysis showed a linear relationship between total and cereal fiber intake with HF prevalence.

Dietary fiber, common nutrition, has a variety of benefits for several diseases (6). A randomized, crossover study documented lowered hyperinsulinemia and plasma lipid concentrations with a high intake of dietary fiber (mainly the soluble type) to improve glycemic control in patients with type 2 diabetes (17). A meta-analysis encompassing 18 studies and 672,408 participants was indicative of an inverse association between dietary fiber consumption and coronary heart disease risk (18). The risk of stroke was lowered by 60% in an 8-year follow-up study with Japanese patients with type 2 diabetes who were in the fourth quartile of total dietary fiber as opposed to those in the first quartile (19). In a large prospective study with a long follow-up period (16.8 years), an inverse association emerged between all-cause mortality and dietary fiber (20). However, the association between HF and dietary fiber intake was not probed in any such work. Our study investigated this association to reveal a lower HF prevalence with higher total and cereal fiber intakes in a dose-responsive manner.

This study found a linear relationship between total fiber intake and HF. HF risk decreased with the increase in total fiber intake, and the rate reduction slightly plateaued at a consumption of 0.3 g/kg/day of total fiber. Similarly, we found a similar dose-response pattern for cereal fiber intake, the rate reduction plateaued at about 0.2 g/kg/day. This observation is consistent with a previous study demonstrating that the risk of hypertension gradually decreased as total dietary fiber intake increased to 0.35 g/kg/day (21). The dose-response relationship in our study suggests the protective role of dietary fiber intake in reducing HF risk, especially for those who do not meet the recommended intake.

The mechanism by which dietary fiber intake might impact HF remains to be elucidated in detail. Several potential pathophysiological mechanisms may contribute to the association between dietary fiber and HF. First, reductions in blood cholesterol and glucose might be involved in the inverse association between dietary fiber intake and HF (22). A study showed that the β-glucans, derived from dietary fiber, lower bile acid reabsorption which in turn reduces the levels of circulating cholesterol (23). The entrapment of sugar by soluble fiber in the small intestine to form a barrier inhibiting amylase and slower glucose absorption is known to diminish blood glucose and improve insulin sensitivity (24). Second, dietary fiber has been shown to improve intestinal flora and increase colonic fermentation (production of SCFA) in the large bowel (25, 26). Animal models have documented that high dietary fiber can improve intestinal flora and reduce blood pressure, cardiac fibrosis, left ventricular hypertrophy, and delay the process of HF in hypertensive mice (26, 27). In addition, other studies reported that high consumption of dietary fiber increased the abundance of SCFA, which might decrease risk factors for HF, such as insulin resistance (28), chronic inflammation, and metabolic disorders (29). The amalgamation of epidemiological and experimental analyses on dietary fiber facilitates us to propose that increasing the dietary fiber intake can cause betterment in HF.

Our study has important clinical implications. Given the increasing prevalence and disease burden of HF along with the lack of approved pharmacological treatment, it is important to scour for modifiable risk factors and to develop preventive strategies. Previous studies have found significant relationships between dietary nutrients and HF (30, 31). Our findings support a potentially adverse association between total, cereal dietary fiber and HF prevalence. The drafting of questionnaires on dietary fiber intake can help to identify individuals at high risk for HF. These observations emerge to reinforce the current recommendations of increasing dietary fiber consumption as part of a healthy diet to prevent HF.

Our study has several advantages. Our study included a large population of different races, social backgrounds, and geographical areas to assess the association between dietary fiber intake and the prevalence of HF. This allowed us to investigate the relationship between dietary fiber and HF for people from different demographics and social backgrounds. However, a few limitations are also put forth here. First, drawing inferences for causal interpretations is challenged towing to the cross-sectional model of our work. Second, despite controlling several potential confounding factors, the possibility of unmeasured confounding factors causing residual confusion remains. Third, the association between specific fiber types (i.e., soluble and insoluble fiber) and HF was not probed which may impact the HF risk.

## Conclusion

A higher intake of total and cereal dietary fiber was associated with a lower prevalence of heart failure in adults, in a dose-response manner. These findings provide further support for the current recommendations that promote increased consumption of dietary fiber to prevent HF.

## Data Availability Statement

The datasets presented in this study can be found in online repositories. The names of the repository/repositories and accession number(s) can be found in the article/supplementary material.

## Ethics Statement

This study was reviewed and approved by the National Center for Health Statistics Research Ethics Review Board, and written informed consent was obtained from all NHANES participants.

## Author Contributions

HZha and ZL contributed to conception and design of the study. DG, HZho, and ZM acquired the data. XZ, YX, and XW performed the statistical analysis. JC and QZh wrote the first draft of the manuscript. QZe and DX critically revised the manuscript. All authors read and approved the final manuscript.

## Conflict of Interest

The authors declare that the research was conducted in the absence of any commercial or financial relationships that could be construed as a potential conflict of interest. The handling editor YH declared a shared affiliation with the authors at the time of review.

## Publisher’s Note

All claims expressed in this article are solely those of the authors and do not necessarily represent those of their affiliated organizations, or those of the publisher, the editors and the reviewers. Any product that may be evaluated in this article, or claim that may be made by its manufacturer, is not guaranteed or endorsed by the publisher.
